# Cholangitis Definition and Treatment after Kasai Hepatoportoenterostomy for Biliary Atresia: A Delphi Process and International Expert Panel

**DOI:** 10.3390/jcm11030494

**Published:** 2022-01-19

**Authors:** Ana M. Calinescu, Omid Madadi-Sanjani, Cara Mack, Richard A. Schreiber, Riccardo Superina, Deirdre Kelly, Claus Petersen, Barbara E. Wildhaber

**Affiliations:** 1 Division of Child’s and Adolescent’s Surgery, Swiss Pediatric Liver Center, Geneva University Hospitals, University of Geneva, 1205 Geneva, Switzerland; barbara.wildhaber@hcuge.ch; 2 Department of Pediatric Surgery, Hannover Medical School, 30625 Hannover, Germany; madadi-sanjani.omid@mh-hannover.de (O.M.-S.); Petersen.Claus@mh-hannover.de (C.P.); 3 Section of Gastroenterology, Hepatology and Nutrition, Digestive Health Institute, Children’s Hospital Colorado, University of Colorado School of Medicine, Aurora, CO 80011, USA; cara.mack@childrenscolorado.org; 4 Division of Gastroenterology, Hepatology and Nutrition, BC Children’s Hospital, University of British Columbia, Vancouver, BC V5Z 4H4, Canada; rschreiber@cw.bc.ca; 5 Division of Transplant Surgery, Ann and Robert H. Lurie Children’s Hospital of Chicago, Northwestern University Feinberg School of Medicine, Chicago, IL 60611, USA; RSuperina@luriechildrens.org; 6 Liver Unit, Birmingham Women’s and Children’s Hospital, Birmingham B15 2TG, UK; deirdrekelly@nhs.net

**Keywords:** biliary atresia, cholangitis, Kasai, hepatoportoenterostomy

## Abstract

(1) Background: Acute cholangitis during the first year after Kasai hepatoportoenterostomy (HPE) has a negative impact on patient and native liver survival. There are no consistent guidelines for the definition, treatment, and prophylaxis of cholangitis after HPE. The aim of this study was to develop definition, treatment, and prophylaxis guidelines to allow for expeditious management and for standardization in reporting. (2) Methods: the Delphi method, an extensive literature review, iterative rounds of surveys, and expert panel discussions were used to establish definition, treatment, and prophylaxis guidelines for cholangitis in the first year after HPE. (3) Results: Eight elements (pooled into two groups: clinical and laboratory/imaging) were identified to define cholangitis after HPE. The final proposed definitions for suspected and confirmed cholangitis are a combination of one element, respectively, two elements from each group; furthermore, the finding of a positive blood culture was added to the definition of confirmed cholangitis. The durations for prophylaxis and treatment of suspected and confirmed cholangitis were uniformly agreed upon by the experts. (4) Conclusions: for the first time, an international consensus was found for guidelines for definition, treatment, and prophylaxis for cholangitis during the first year after Kasai HPE. Applicability will need further prospective multicentered studies.

## 1. Introduction

Acute cholangitis after Kasai hepatoportoenterostomy (HPE) is known to have a negative impact on prognosis; it predicts liver failure [1] and is associated with earlier liver transplantation [2]. Furthermore, repeated cholangitis episodes are thought to be an important factor contributing to the progression of liver cirrhosis, ultimately leading to liver transplantation in biliary atresia patients and to decreased survival rates [3,4,5,6,7].

Reports on the incidence of cholangitis in biliary atresia patients vary between 40% and 93% [8]. Most of the cholangitis episodes develop within the first two years of life, and especially within the first year of life [9,10,11,12]. Despite improvements in postoperative management over the last decades, the incidence of cholangitis remains stable over time [13]. Hypotheses about the etiology of cholangitis includes intestinal bacterial migration, translocation from lymphatics, hematogenous spread via portal vein as well as an immune inflammatory response [14]. While attempts have been made to standardize the diagnosis of cholangitis after Kasai HPE, there are still no clear guidelines as to how to define the disease [15]. The Tokyo Guidelines, developed for adult patients, are clearly not applicable to diagnose cholangitis in children during the first year after Kasai HPE [16,17].

The use of prophylactic antibiotics has been shown to be beneficial to decrease the rate of recurrent cholangitis [18]. However, prophylaxis must be balanced against the possibility of lethal cholangitis secondary to resistant organisms [4]. This said, it is almost impossible to compare the existing body of literature due to the wide variety in practices of cholangitis prophylaxis and, again, the lack of a unanimous thus comparable definition of cholangitis [5,19].

Quick and effective treatment of cholangitis after Kasai HPE is paramount. The threshold for suspecting cholangitis must be low, allowing for the introduction of a prompt and effective treatment to avoid further liver damage as well as potentially lethal septicemia. For prophylaxis, antibiotic regimens and durations are widely variable in the pediatric literature [10,20,21].

The aim of this work was to propose unambiguous criteria for the diagnosis and treatment of cholangitis after Kasai HPE for biliary atresia patients during the first year after Kasai HPE, based on a systematic review of the literature and the consensus of international experts, reached within the Biliary Atresia and Related Disorders (BARD) community (http://www.bard-online.com/, accessed on 15 December 2021) and during a Webinar held in July 2021.

## 2. Materials and Methods

### 2.1. Systematic Literature Review

We systematically reviewed the following databases: Embase, PubMed, Web of Science, and the Cochrane Database from the beginning of each database through November 2019. We used the search terms: “Cholangitis”(Mesh:noexp) OR Cholangitis(Title/Abstract) AND (“Portoenterostomy, Hepatic”(Mesh) OR “Biliary Atresia”(Mesh) OR “Hepatic Portoenterostomy”(Title/Abstract) OR “Hepatic Portoenterostomies”(Title/Abstract) OR hepatoportoenterostomies(Title/Abstract) OR hepatoportoenterostomy(Title/Abstract) OR “Kasai Procedure”(Title/Abstract) OR “Kasai portoenterostomy”(Title/Abstract) OR “Post-Kasai”(Title/Abstract) OR “Kasai operation”(Title/Abstract) OR biliary atresia(Title/Abstract)). Two authors (AMC and OMS) completed the search strategy independently. Selected titles and abstracts were reviewed to identify suitable articles that gave information about definition and/or antibiotic prophylaxis and/or antibiotic treatment of cholangitis after Kasai HPE. Whether studies met the eligible criteria was determined based on author consensus. Language was restricted to English. Systematic literature review set the base for the 1st Delphi questionnaire for the definition and treatment of cholangitis.

### 2.2. Formatting and Pretesting of the 1st Delphi Questionnaire

To establish the different consensus, the well-structured Delphi method was used as proposed by Dalkey N.C. [22,23].

*Study design 1st Delphi questionnaire*: self-administered, web-based survey using the online tool SurveyMonkey (http://www.surveymonkey.com, accessed on 13 June 2021).

*Study outcomes 1st Delphi questionnaire*: Study outcomes were stated as: (i) to define items included in the cholangitis definition (primary outcome) and (ii) to identify current practices for primary prophylaxis after HPE and treatment of cholangitis occurring in biliary atresia patients within the first year after HPE (secondary outcomes). Of note, the terms cholangitis and acute cholangitis were used interchangeably throughout the questionnaires and the manuscript.

*Study population 1st Delphi questionnaire*: The survey targeted pediatric surgeons and hepatologists working in Europe, North America, Asia, and Australia. The questionnaire was electronically distributed to the 34 faculty members of BARD and 28 centers of the European Reference Network—Rare Liver.

*Development of the 1st Delphi questionnaire:* The variables assessed in the 1st Delphi questionnaire (regarding cholangitis definition, primary prophylaxis after Kasai HPE, and treatment) were selected with the help of the systematic literature review and by consulting international experts in biliary atresia. The questionnaire was initiated using a semi-structured interview, separately run with two experienced pediatric surgeons, with the aim of identifying redundant, irrelevant, or poorly worded questions [24]. Clinical sensibility testing of the questionnaire, aiming to assess its comprehensiveness, clarity, and validity was then conducted by running the questions to 10 other pediatric surgeons and hepatologists to be answered with a 7 point Likert scale. Finally, the reliability of the questionnaire was assessed with a test re-test: the questionnaire was given to the same 10 pediatric surgeons and hepatologists after a 2 week interval, and the reproducibility of their answers was assessed with a Spearman correlation coefficient (0.73). The survey was held in English. No questions were mandatory; each participant could advance in the survey after skipping a question. The questionnaire is depicted in Supplementary Materials Document S1.

*Distribution of the 1st Delphi questionnaire*: The survey was distributed by e-mail, with a cover letter stating the objectives of the survey and providing an estimate of the completion time, according to the principles of Dillman and recommendations of Burns and coworkers [24]. The first e-mail was sent in August 2020, and two reminder e-mails were sent two and four weeks later.

### 2.3. Format of the 2nd Delphi Questionnaire

*Study design 2nd Delphi questionnaire*: idem. 1st questionnaire.

*Study outcomes 2nd Delphi questionnaire*: While the 1st questionnaire allowed for identification of criteria to use in the definition of acute cholangitis, this 2nd questionnaire aimed at (i) confirming the weighting of individual criteria in order to provide consensus for a cholangitis definition after Kasai HPE and (ii) to define the regimen and duration of primary prophylaxis after Kasai HPE and treatment of cholangitis. Thus, study outcomes were stated as: (i) to define biliary atresia-associated cholangitis (primary outcome) and (ii) to define biliary atresia-associated cholangitis prophylaxis and treatment (secondary outcomes).

*Study population 2nd Delphi questionnaire*: The survey targeted pediatric surgeons and hepatologists of the 34 faculty members of BARD only.

*Development of the 2nd Delphi questionnaire*: Criteria that achieved a consensus of more than 50% of the Delphi 1 participants (1st questionnaire) were taken into consideration for the 2nd Delphi questionnaire. As in clinical practice we often suspect cholangitis in infants after HPE and start treatment even if cholangitis is not yet confirmed, we stratified definitions of cholangitis in (i) suspected and (ii) confirmed, each of them with a respective duration of antibiotic treatment. Further, regimen and duration of primary prophylaxis after Kasai HPE was addressed. The questionnaire was administered to three experienced pediatric surgeons with the aim of identifying redundant, irrelevant, or poorly worded questions [24]. The questionnaire is available in Supplementary Materials Document S2.

*Distribution of the 2nd Delphi questionnaire*: idem. 1st questionnaire.

*Administration of the 2nd Delphi questionnaire*: idem. 1st questionnaire. The first e-mail was sent in April 2021, with a reminder e-mail two weeks later.

### 2.4. Pre-Meeting Working Group

A working group of 3 hepatologists (CM, RSc, and DK) and 5 surgeons (AMC, OMS, RSu, CP, and BEW) analyzed the results from the 2nd Delphi questionnaire and unanimously agreed on a proposed definition for suspected and confirmed cholangitis, treatment of suspected and confirmed cholangitis, and for prophylaxis of primary cholangitis after Kasai HPE. This process took place from 18 June through 22 June 2021.

### 2.5. Expert Panel Meeting

The proposed definitions of suspected and confirmed cholangitis, primary prophylaxis after Kasai HPE, and treatment of suspected and confirmed cholangitis were discussed within an expert panel meeting during the BARD Webinar held on 1 July 2021 as well as with the other participants in the webinar through a live chat. Panelists were provided with a summary depicting the rankings from the 1st and 2nd Delphi survey as well as the pre-meeting working group proposal. BEW served as moderator of the meeting. Approximatively 10 min of open-ended discussion was allotted for each of the three matters. The chat discussions and the recording of the webinar were used to capture the key elements and the discussion topics.

### 2.6. Statistical Analysis

The Pearson correlation coefficient was used to indicate correlation between the 1st and 2nd Delphi questionnaires.

## 3. Results

### 3.1. Literature Search

The literature search identified 615 scientific papers, and 109 publications finally met the inclusion criteria and were selected for full article review.

The clinical definition of cholangitis used in the literature varied largely (Appendix A, Table A1). The following items were used to define cholangitis: fever in 73.3% (80/109) of the studies; new or increasing jaundice was used in 55% (60/109); fever *and* new or increasing jaundice in 33.9% (37/109); stool color change in 44.9% (49/109); some form of abdominal discomfort in 6.4% (7/109) of the selected articles.

The laboratory elements for defining cholangitis were elevated bilirubin in 60.5% (66/109); white blood cells and elevated liver tests in 32.1% (35/109); two laboratory criteria (elevated white blood cells (WBCs) and elevated bilirubin) in 29.3% (32/109), elevated inflammatory parameters (C-reactive protein (CRP) and/or procalcitonin (PCT)) in 16.5% (18/109); positive blood cultures were required to define cholangitis in 13.7% (15/109) of the articles.

The presence of bile lakes was included in the definition of cholangitis for 12.8% (14/109) of the authors.

The most frequently administered antibiotic prophylaxis was sulfamethoxazole and trimethoprim in 39.4% (15/38) of the articles (Table 1). Primary antibiotic prophylaxis (after the immediate postoperative period) was given between 6 and 12 months in 29.4% (5/17) of the reviewed articles.

The most common antibiotic for the treatment of cholangitis was ceftriaxone in 51.6% (16/31) of the studies for a duration of 2 weeks in 46.1% (6/13) of the reviewed articles (Table 2).

### 3.2. 1st Delphi Questionnaire

**Figure 1:**
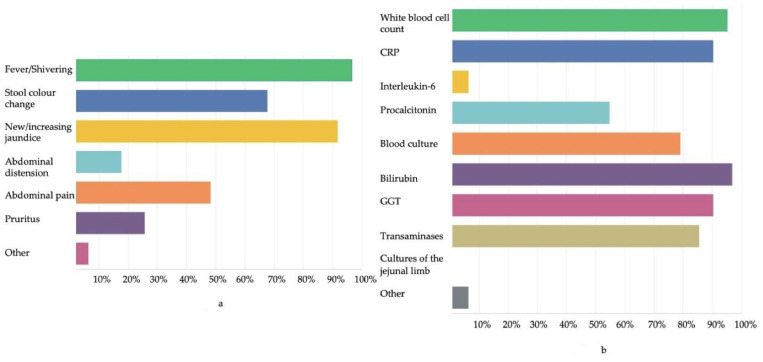
First Delphi questionnaire (**a**) Clinical signs following Kasai hepatoportoenterostomy included in the definition of cholangitis according to the 1st Delphi questionnaire. (**b**) Laboratory and imaging elements following Kasai hepatoportoenterostomy included in the definition of cholangitis according to the 1st Delphi questionnaire.

The 1st Delphi questionnaire was answered by 62 surgeons and hepatologists. Clinical elements defining cholangitis were answered as follows: fever/shivering 96.7% (60/62), stool color change 67.4% (42/62), new or increasing jaundice 91.9% (57/62), and abdominal distension/abdominal pain 66.1% (41/62) (Figure 1a). Laboratory elements defining cholangitis included increased levels of WBCs 95.1% (59/62), CRP 90.3% (56/62), PCT 54.8% (34/62), bilirubin 96.7% (60/62), gamma-glutamyl transferase (GGT) 90.3% (56/62), transaminases 85.4% (53/62), and positive blood cultures 79% (49/62). Bile lakes were included in the definition of cholangitis by 63.3% of the participants (43/62) (Figure 1b).

Of the participants, 89.6% (52/62) answered affirmatively with regard to primary antibiotic prophylaxis to prevent cholangitis after HPE; 53.8% (28/62) declared using sulfamethoxazole–trimethoprim.

The duration of cholangitis treatment was answered as 3 weeks according to 29% (16/62) of the participants, with piperacillin–tazobactam in 70.9% (39/62) of the answers.

### 3.3. 2nd Delphi Questionnaire

The response rate of the 2nd Delphi questionnaire was 44.1% (15/34).

The clinical elements included in the definition of cholangitis showed a Pearson correlation coefficient of 0.9 (*p* = 0.004) between the 1st and 2nd Delphi questionnaires. We did not correlate the laboratory and imaging elements between the two surveys, as items from the 1st Delphi survey were merged into fewer elements in the 2nd Delphi survey.

In the 1st Delphi survey, eight elements were identified as defining cholangitis and were pooled in two groups: (A) clinical elements—fever without extrahepatic source and/or shivering, stool color change, new/increasing jaundice, abdominal discomfort (vomiting, poor feeding, and irritability); (B) laboratory and imaging elements—inflammatory response (WBCs and/or CRP and/or PCT), increased/increasing transaminases, increased/increasing GGT and/or bilirubin, and bile lakes.

**Figure 2:**
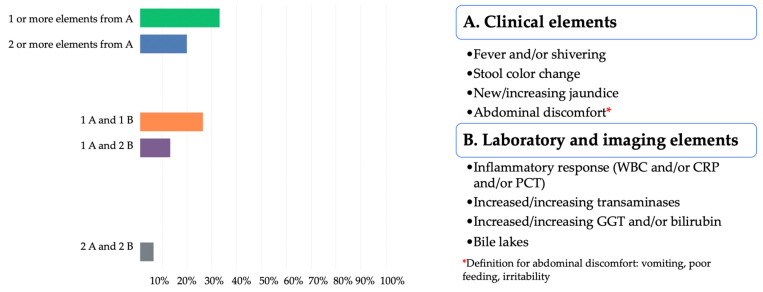
Definition of suspected cholangitis according to the 2nd Delphi questionnaire.

As for the definition of suspected cholangitis, the participants identified mainly one or more elements from A 5/15 (33.3%) and one element from A and one element from B 4/15 (26.67%) (Figure 2).

**Figure 3:**
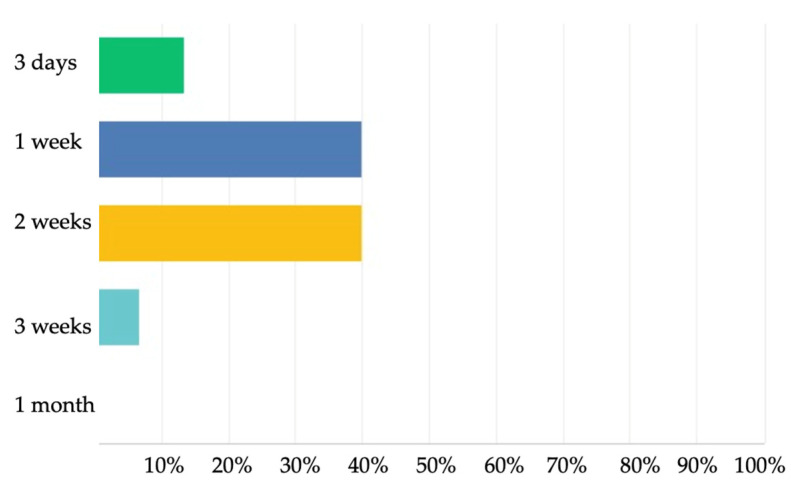
Treatment duration of suspected cholangitis according to the 2nd Delphi questionnaire.

The treatment duration of a suspected cholangitis was selected to be 1 or 2 weeks by 6/15 (40%) of the participants (Figure 3).

**Figure 4:**
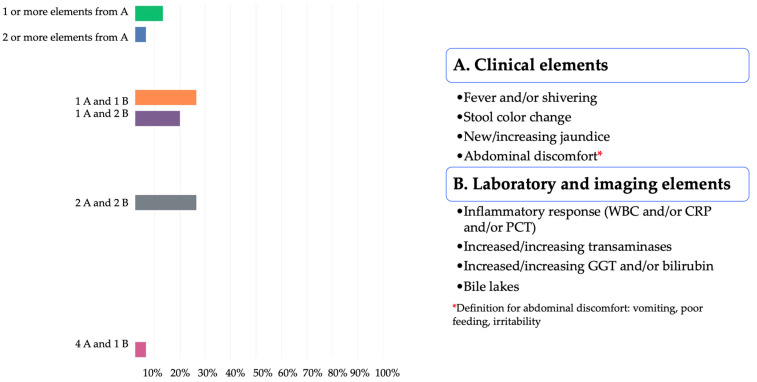
Definition of confirmed cholangitis according to the 2nd Delphi questionnaire.

As for the definition of confirmed cholangitis, the participants identified equally one element from A and one element from B and two elements from A and two elements from B, 4/15 (26.67%) both choices (Figure 4).

**Figure 5:**
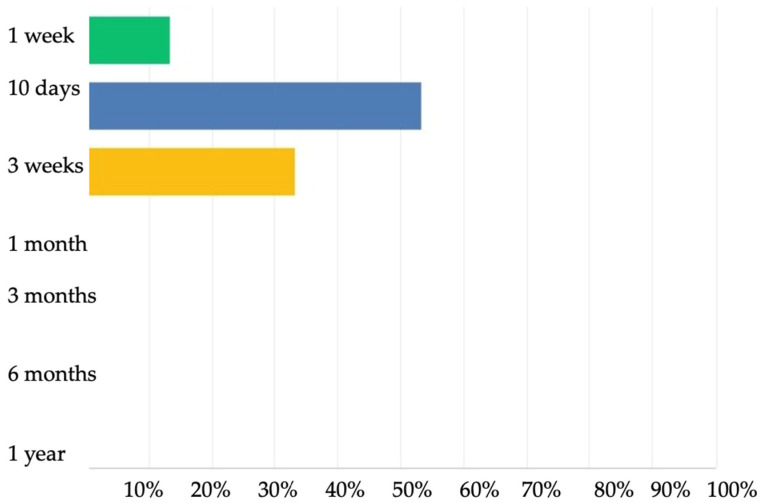
Treatment duration of confirmed cholangitis according to the 2nd Delphi questionnaire.

The treatment duration of a confirmed cholangitis was selected to be 10 days for 8/15 (53.3%) and 3 weeks for 5/15 (33.3%) (Figure 5).

**Figure 6:**
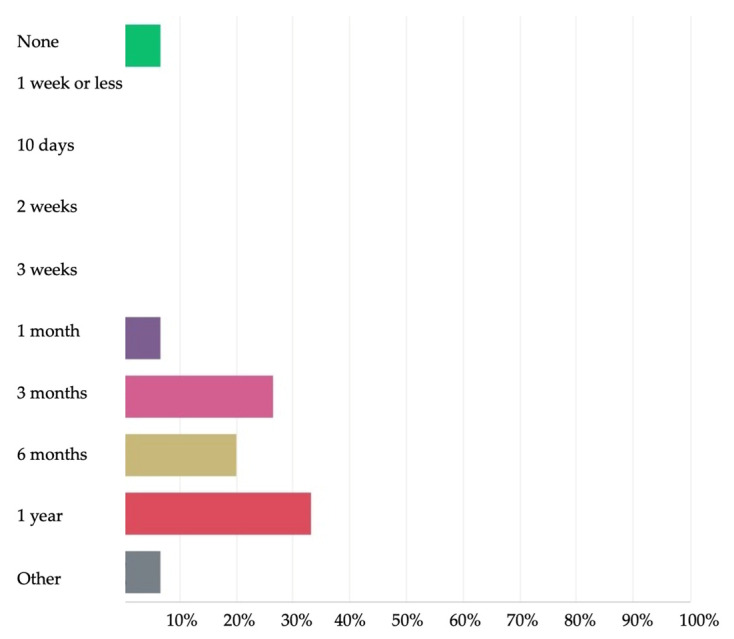
Peroral prophylaxis duration after Kasai hepatoportoenterostomy according to the 2nd Delphi questionnaire.

The duration of the peroral primary prophylaxis after HPE was most frequently answered to be 1 year by 5/15 (33.3%) of the participants and 3 months by 4/15 (26.6%) (Figure 6).

For both treatment duration and prophylaxis, the correlation coefficient was not applicable due to the modified subgroups between the two Delphi surveys.

### 3.4. Pre-Meeting Working Group

**Figure 7:**
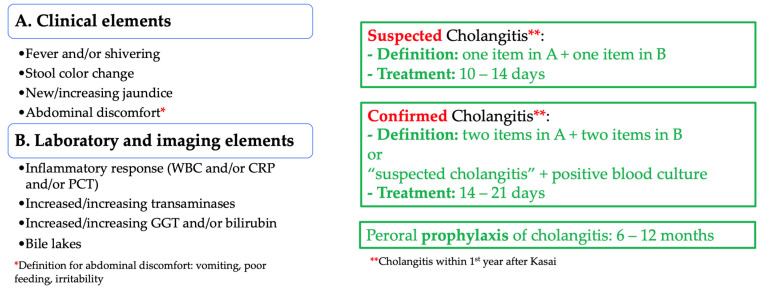
Working group proposal for cholangitis definitions, prophylaxis, and treatment.

The working group analyzed the results from the 1st and 2nd Delphi surveys and an agreement was found as described in Figure 7. The chosen definitions for suspected and confirmed cholangitis were based on a combination of the eight clinical, laboratory, and imaging items. Of note, the working group added that a suspected cholangitis having a positive blood culture transformed into a confirmed cholangitis.

### 3.5. Expert Panel

Comments from the expert panel meeting are summarized in Section 4.

## 4. Discussion

Cholangitis, a potentially life-threatening condition after the Kasai HPE, is defined as inflammation or infection of the bile duct system [45]. Although the definition of the pathological picture of cholangitis is unequivocal, diagnostic criteria are far from clear-cut, and many different definitions exist to delineate the clinical diagnosis. Whereby some clinicians suspect post-Kasai cholangitis in any situation where the patient is “not well”, others need a clearly febrile baby to feel in line with the diagnosis. When reviewing the literature on the topic, this discrepancy between different definitions is immediately depictable, mirroring the difficulties clinicians have to diagnose their patients. This study aimed to find, via the Delphi method and an expert panel, a consensus on the criteria that have diagnostic importance for cholangitis after Kasai HPE, thus defining suspected and confirmed cholangitis. Further, we established recommendations for cholangitis prophylaxis and a treatment plan for each suspected and confirmed cholangitis.

### 4.1. Definitions of Suspected and Confirmed Cholangitis

In the pediatric literature few authors discuss the concept of a suspected or presumed cholangitis [21,46], but terms such as *suspected* and *definite* diagnosis appear within the Tokyo guidelines, which guide the clinician to the diagnosis of cholangitis [16]. Yet, regarding the clinical applicability of the Tokyo guidelines, it is important to note that they have been tested only in adult cohorts [47]. Thus, there is a consensus among experts that these guidelines clearly do not seem suitable for small children. Based on the Tokyo guidelines our pediatric expert panel intensively discussed the weight of the included items for a definition in young children. Although some items were unanimously supported, such as fever or shivering and sudden stool color change as well as inflammatory laboratory elements, some needed extensive discussions. This said, the Delphi method clearly helped to weigh the different opinions and to come to a consensus. Of note, the idea to create a score by attributing a value for each item and to define cholangitis when a certain total value is reached was rapidly rejected due to the (1) lack of evidence and (2) the more difficult implementation and, thus, the less likelihood to be used in the everyday clinical practice.

Of note, the following definitions are proposed for first episode(s) of cholangitis within the first year after Kasai HPE and are not thought to be used to define refractory and/or recurrent cholangitis 1 year after Kasai HPE.

*Suspected cholangitis*: The definition for *suspected* cholangitis that was finally chosen was **one** item from the list A (fever and/or shivering, *or* stool color change, *or* new/increasing jaundice, *or* abdominal discomfort) and **one** item from the list B (inflammatory response, *or* increased/increasing transaminases, *or* increased/increasing GGT or bilirubin, *or* the presence of bile lakes) to define this clinical picture needing the related treatment (Figure 5). Both the working group and expert panel agreed to have a very low threshold to suspect cholangitis in order for babies not to be missed and potentially evolve towards severe, life-threatening confirmed cholangitis. Swift and prompt cholangitis treatment in this circumstance might avoid liver deterioration.

*Confirmed cholangitis*: The proposed definition for *confirmed* cholangitis included **two** items from list A and **two** items from list B (Figure 5). Further, the expert panel proposed that a baby with *suspected* cholangitis revealing a positive blood culture should shift the diagnosis to confirmed cholangitis. Of note, reported rates of positive blood cultures in cholangitis were variable: according to the published series, they ranged from 25.8% to 43.6% [12,48]. A further consideration from the panelists was to include the treatment response of a suspected cholangitis into the definition of a confirmed cholangitis but decided that this should finally be up to the discretion of the treating clinician and should not be included in the definition. Although participants and experts initially were positive to include fever *without* an extrahepatic source as a mandatory criterion for a confirmed cholangitis, we concluded that two *other* additional clinical criteria from the list A, associated with two laboratory elements, can also confirm cholangitis. Some experts and participants also proposed liver biopsy (percutaneous or laparoscopic) to confirm cholangitis. Although the risk for major complications associated with liver biopsy has been reported to be less than 1% [49], the procedure is invasive, and should be limited to specific clinical situations such as intractable cholangitis without positive blood cultures or recurrent cholangitis [48,50]. The item was therefore not included in the definition of confirmed cholangitis in the discussed setting, i.e., children within the 1st year after Kasai HPE who present with a first episode of cholangitis.

### 4.2. Treatment of Suspected and Confirmed Cholangitis

*Suspected cholangitis*: The treatment duration for a suspected cholangitis was unanimously preferred to be 10–14 days. This recommendation is in line with the shorter antibiotic treatment duration for post Kasai HPE cholangitis reported in the literature [5,10,13,40]. Both working group and expert panel agreed that this proposed treatment duration is adequate for patients with the diagnosis of suspected cholangitis, but also agreed that a treatment duration of only 7–10 days seems too short and risks leading to episodes of recurrent or refractory cholangitis. Whether 10 or 14 day therapy is chosen is up to the discretion of the treating clinician and the level of suspicion for suspected cholangitis.

*Confirmed cholangitis*: The treatment duration for confirmed cholangitis was chosen to be 14–21 days, corresponding to the reported longer treatment duration for cholangitis after Kasai HPE [21,43]. The choice between a 14 and 21 day treatment regimen is up to the clinician, who will decide based on the patient’s clinical condition and treatment response. The working group and panel participants both readily agreed on this treatment duration and there was no debate on this subject.

### 4.3. Cholangitis Prophylaxis

In the literature, there is uncertainty regarding long-term prescription of antibiotics to prevent cholangitis after Kasai HPE; little published data support the use of one antibiotic over another [51] or a specific duration of antibiotic prophylaxis [8]. Further, the fact that antibiotic prophylaxis might induce antibiotic resistance must also be taken into consideration. Yet, as outlined by the overwhelming majority of the respondents of our surveys and the expert panel, clinical practice favors antibiotic administration to prevent cholangitis after Kasai HPE. Weighing the benefits and risks, the participants and expert panel chose the duration of prophylaxis to be 6–12 months. Of note, no differentiation was suggested to be made between draining and non-draining HPE.

## 5. Conclusions

We herein have developed standardized definitions for suspected and confirmed cholangitis after Kasai HPE. The definitions include the most important clinical, laboratory, and imaging criteria for cholangitis, identified through a group of international experts using the Delphi method. These definitions can not only be easily applied in the clinical setting of non-specialized, general pediatric clinics, but may also be used as an outcome measure in studies reporting on complications after Kasai HPE and/or the impact of cholangitis on native liver survival and patient survival. The duration of antibiotic prophylaxis and treatment was identified in the literature review and confirmed by both Delphi survey participants and panelists. Preliminary applicability will be further tested in a multicentered prospective study.

## Figures and Tables

**Table 1:** Overview of reported (2000–2021) cholangitis prophylaxis after Kasai hepatoportoenterostomy and cholangitis rates. Bid, bis in die; Qid, quater in die.

Authors	Nr. px	Cholangitis Prophylaxis	Cholangitis Prophylaxis Duration	Cholangitis Rates
Chuang J., et al., 2000 [13]	39	Sulfamethoxazole	3 months	46%
Lally K.P., et al., 1989 [25]	41	Sulfamethoxazole; Ampicillin; Cephalosporins	1 to several months	21.9%
Wu E.T., et al., 2001 [10]	37	Sulfamethoxazole 4 mg/kg or Neomycin 25 mg/kg 4×/week	Unknown	75%
Bu L.N., et al., 2003 [9]	19	Sulfamethoxazole 20 mg/kg/d bid or Neomycin 25 mg/kg/d qid, 4 days/week	6–7 months	-
Meyers R.L., et al., 2003 [26]	28	Piperacillin/Tazobactam 300 mg/kg/d qid + Gentamycin 5 mg/kg/d or Cefoperazone 150 mg/kg/d divided into 3 doses followed by Sulfamethoxazole 10 mg/kg/d bid	First regimen given 2–3 months and then unknown	34.4%
Lai H.S., et al., 2006 [18]	163	Sulfamethoxazole 20 mg/kg/d bid or Neomycin 25 mg/kg/d, qid, 4 days/week	3 years	72.3%
Hung P.Y., et al., 2006 [4]	185	Oral antibiotics	1–6 months	54.6%
Kelly D.A., et al., 2007 [27]	-	Amoxicillin or Cephalexin or Sulfamethoxazole	Alternate every 2–3 months for 1 year minimum	-
Stringer M.D., et al., 2007 [28]	71	Cephalexin 25 mg/kg 2×/day oral	1 month	46%
Vejchapipat P., et al., 2007 [29]	53	Cotrimoxazole	1 year	45.2%
Petersen C., et al., 2008 [21]	49	Cefaclor 45 mg/kg/d oral	1 year	-
De Vries W., et al., 2012 [30]	214	Sulfamethoxazole or Neomycin/Colistin/Nystatin or Ciprofloxacin	-	55.1%
Wang B., et al., 2014 [31]	25	-	6 months	35%
Tyraskis A., et al., 2016 [32]	104	Cefalexin 25 mg/kg/d	1 month	-
Webb N.L., et al., 2016 [33]	29	-	>1 year	75%
Lee W.S., et al., 2017 [34]	52	-	3 months	36%
Pang W., et al., 2019 [19]	218	3rd generation Cephalosporin, oral	6 months	27%
Parolini F., et al., 2019 [35]	174	Sulfamethoxazole and Cephalosporin, 1 year if good bile drainage	1 year	32%
Ramachandran P., et al., 2019 [36]	62	Alternating Amoxicillin–Clavulanic Acid 40 mg/kg/d bid and Cefpodoxime 10 mg/kg/d bid, alternating	6 months	43.5%
Baek S.H., 2020 [37]	160	None	None	78.8%
Chen G., et al., 2021 [38]	180	Sulfamethoxazole 25 mg/kg/d bid for 2 weeks then Cefaclor 40 mg/kg/d bid for 2 weeks, alternating every 2 weeks	6 months	66.1%
Goh L., et al., 2021 [39]	54	Cotrimoxazole	1 year minimum	72%

**Table 2:** Overview of reported (2000–2021) cholangitis treatment after Kasai hepatoportoenterostomy and native liver survival rates if available. NLS, native liver survival; CRP, C-reactive protein.

Authors	Number of Patients	Cholangitis Treatment	Cholangitis Treatment Duration	Native Liver Survival
Chuang J., et al., 2000 [13]	39	Cephalosporin and Aminoglycoside	7–10 days or till negative CRP	-
Wu E.T., et al., 2001 [10]	37	Ceftriaxone	At least 5 days	-
Van Heurn E., et al., 2003 [14]	77	3rd generation Cephalosporin	1 week	-
Wong K.K., et al., 2004 [20]	19	Cefoperazone 25 mg/kg 3×/day or Meropenem 20 mg/kg 3×/day	2 weeks	-
Petersen C., et al., 2008 [21]	49	3rd generation Cephalosporin and Aminoglycoside	3 weeks	6 month, NLS 63% 2 year, NLS 31%
Lee J Y., et al., 2014 [12]	27	Ampicillin, Gentamycin, and Metronidazole or Unasyn	14 days	-
Lien T., et al., 2015 [40]	20	Ceftriaxone	14 days	-
Chiang L.W., et al., 2017 [41]	58	Ceftriaxone 100 mg/kg/day or Piperacilline–Tazobactam 320 mg/kg/day recently	-	Overall NLS, 48.3% 2 year NLS, 72% 5 year NLS, 45.7%
Lee W.S., et al., 2017 [34]	52	-	10–14 days	NLS, 37%
Li Z., et al., 2017 [42]	80	Meropenem or Cefoperazone	-	-
Li D., et al., 2018 [5]	113	Meropenem 20 mg/kg 3×/j	5 days	-
Calinescu A.M., et al., 2019 [43]	62	Piperacillin–Tazobactam	3 weeks	4 year NLS for cholangitis patients, 36%
Ramachandran P., et al., 2019 [36]	62	Piperacillin–Tazobactam	-	1 year NLS for cholangitis patients, 33%
Chung P.H.Y., et al., 2020 [44]	128	Meropenem Cefoperazone	2 weeks 2 weeks	1 year NLS, 85.7% 1 year NLS, 69%

## Supplementary Materials

The following are available online at https://www.mdpi.com/article/10.3390/jcm11030494/s1, Supplementary Document S1: Cholangitis definition and management after Kasai hepatoportoenterostomy for biliary atresia questionnaire—1st Delphi questionnaire; Supplementary Document S2: Definition of cholangitis and management after Kasai hepatoportoenterostomy for biliary atresia—2nd Delphi questionnaire.

## Appendix A

**Table A1:** Overview of cholangitis definition after Kasai hepatoportoenterostomy and reported (2000–2021) rates of cholangitis, native liver survival (NLS), and patient survival (PS). ALT, alanine aminotransferase; AST, aspartate aminotransferase; CRP, C reactive protein; GGT, gamma-glutamyl transpeptidase; NLS, native liver survival; PA, alkaline phosphatase; PS, patient survival; WBC, white blood cell count.

Authors	Number of Patients	Cholangitis Definition	Cholangitis Rates	NLS/PS
Chuang J., et al., 2000 [13]	39	Fever > 38 °C without obvious extrahepatic source with an elevated serum bilirubin	46%	-
Wu E.T., et al., 2001 [10]	45	Fever, acholic stools, and/or increasing jaundice +/− positive blood cultures	75%	PS, 67.5%
Selvalingam S., et al., 2002 [52]	61	Fever and leukocytosis (no other cause) + increase direct bilirubin or AST or ALT or paler stools +/− positive blood culture	57%	1 year PS, 90%
Bu L.N., et al., 2003 [9]	19	Unexplained fever ≥ 38 °C, acholic stools, increased jaundice or positive blood culture	100%	-
Van Heurn E., et al., 2003 [14]	77	Fever > 38 °C, not explained otherwise or abrupt recurrence or increase of clinical jaundice with increased bilirubin levels or acholic stools		
Ogasawara Y., et al., 2003 [53]	21	Fever > 38 °C and elevated bilirubin and leukocytosis	52.3%	PS, 100%
Wong K.K., et al., 2004 [20]	19	Fever > 38.5 °C of unknown origin more than 48 h, progressive jaundice and derangement of liver function, passage of acholic stools	-	-
Kobayashi H., et al., 2005 [54]	63	Fever > 38 °C, with elevated serum bilirubin and leukocytosis	15.8%	-
Shinohara T., et al., 2005 [55]	18	Unexplained fever > 38 °C, with elevated CRP and bilirubin.	44.4%	-
Hung P., et al., 2006 [4]	22	High fever with no other obvious focus with acholic stools, increased jaundice, or positive blood culture	54.6%	2 year NLS, 53.2% 5 year NLS, 34.7% 10 year NLS, 30.5%
Lai H.S., 2006 [18]	163	Recurrent clay colored stool, icterus, or hyperbilirubinemia	72.3%	-
Stringer M.D., et al., 2007 [28]	71	Deteriorating liver function + pale stools and fever	46%	NLS, 67.5% PS, 93.3%
Vejchapipat P., et al., 2007 [29]	53	Fever > 38.5 °C, change of stool color, leukocytosis (>12 G/L) with polymorphonuclear leukocytes predominance	45.2%	-
Petersen C., et al., 2008 [21]	49	Suspected cholangitis: any of fever, recurrence of acholic stools, leukocytosis, elevated liver function tests, increasing bilirubin	-	6 month, NLS 63% 2 year, NLS 31% 6 month, PS 90% 2 year, PS 78%
Sanghai S.R., et al., 2009 [56]	88	Fever with clay colored stool, leukocytosis and/or vomiting, abdominal distension and bacteriemia	33.3%	-
Suzuki T., et al., 2010 [57]	53	Fever, blood biochemistry and the decrease of bile secretion (fecal color change)	13.2% (early)	NLS, 73.6% PS, 88.7%
Kumagi T., et al., 2011 [46]	22	Presumed cholangitis: fever and chills with or without jaundice, nausea or abdominal pain and abnormal biliary imaging: stricture, dilatation and/or stone, with or without evidence of an acute rise in liver tests or improvement upon administration of antibiotics	50%	PS, 95.5% NLS, 81.8%
Lee J.Y., et al., 2014 [12]	27	Fever > 37.5 °C or worsening jaundice, transaminitis or acholic stools +/− positive blood cultures	64.3%	-
Ng V., et al., 2014 [58]	219	Fever > 38 °C without other obvious source, new onset of acholic stools, right upper quadrant pain or tenderness and both elevation of direct bilirubin by 25% and at least 1 mg/dL above baseline, positive blood culture not required	62.1%	-
Wada M., et al., 2014 [59]	36	Elevated serum bilirubin > 2.5 mg/dL, leukocytosis with left shift and normal to acholic stools in a febrile patient (>38 °C)	48.8%	-
Lien T., et al., 2015 [40]	20	Unexplained fever > 38 °C, acholic stools, increased jaundice or positive blood cultures	20%	-
Qiao G., et al., 2015 [60]	262	Fever > 38 °C, without other reason, recurrence or increased jaundice, increased bilirubin, acholic stools	54.9%	5 year PS, 43.3% 5 year NLS, 75.8%
Webb N.L., et al., 2016 [33]	29	Fever > 38.5 °C, and elevated liver transaminases in the absence of other cause for febrile illness	75%	5 year NLS, 45.8%
Chiang L.W., et al., 2017 [41]	58	Fever without other attributable cause, acholic stool and/or deepening jaundice	30.5%	Overall NLS, 48.3% 2 year NLS, 72% 5 year NLS, 45.7%
Kelay A., et al., 2017 [61]	-	Fever, abdominal pain, worsening or recurring jaundice with acholic stools, changes in bilirubin and liver enzymes level together with acute changes in WBC and inflammatory markers such as CRP	-	-
Lee W.S., et al., 2017 [34]	52	Fever > 38 °C without other source, abdominal pain and new onset of acholic stools, and elevation of conjugated bilirubin and/or GGT from previous baseline	52%	NLS, 37% PS, 51%
Stagg H., et al., 2017 [15]	-	Fever and/or jaundice, altered liver biochemistry, blood cultures (96%) and liver biopsy (26%)	-	-
Chen S., et al., 2018 [3]	366	Fever ≥ 38 °C and acholic stool, increase of jaundice and bilirubin or positive blood cultures	67.7%	NLS, 74%
Chung P., et al., 2018 [48]	192	Fever ≥ 38.5 °C, with either increased bilirubin ≥ 20 μmol/L or acholic stool.	35.4%	-
Jiang H., et al., 2018 [62]	-	High fever, bile discharge reduced or stopped, abdominal distention, vomiting and reduced liver function, worsening jaundice, elevated levels of Bilirubin and ALAT, pale or clay-colored stools, dark yellow colored urine, WBC and neutrophils elevated	-	-
Li D., et al., 2018 [5]	113	Fever without identifiable source and 1. Reappearance of jaundice or acholic stools; 2. Sudden elevation of bilirubin > 2.5 mg/dL or AST or 3. Positive blood culture	-	-
Nakajima H., et al., 2018 [63]	66	Fever > 38 °C, elevated serum bilirubin > 2.5 mg/dL, leukocytosis with left shift and normal to acholic stools	55%	NLS, 74%
Xiao H., et al., 2018 [64]	166	Fever > 38 °C, unexplained by other reasons, abrupt recurrence or increased clinical jaundice with increased bilirubin levels, acholic stools, significantly increased serum WBC and neutrophil	44.5%	2 year NLS, 79.5%
Ginstrom D., et al., 2019 [65]	61	Fever > 38 °C without any other identifiable source, treated with intravenous antibiotics	79%	-
Liu J., et al., 2019 [6]	180	At least 2 of: 1. Unexplained fever > 38 °C, 2. Recurrence or exacerbation of jaundice with increased bilirubin or changes from yellow to acholic stools, 3. Elevated CRP	66.1%	NLS, 53.9% PS, 80%
Pang W., et al., 2019 [19]	218	Fever and/or altered stool or refractory jaundice, CRP and/or WBC elevation and sudden elevation of bilirubin or ALT or AST	27%	-
Parolini F., et al., 2019 [35]	174	Fever, abdominal pain, worsening or recurrent jaundice, change in stool color associated with rise in bilirubin and liver enzyme levels, white cell count and inflammatory markers	32%	20 year NLS, 18.3%
Ramachandran P., et al., 2019 [36]	62	1. Fever, pale stools. 2. Elevated WBC and CRP. 3. Elevation of bilirubin and/or liver enzymes	43.5%	-
Baek S.H., et al., 2020 [37]	160	Fever > 38 °C or elevated inflammatory markers and evidence of cholestasis or abnormal liver function tests in accordance with Tokyo guidelines	78.8%	5 year PS, 93.3%
Madadi-Sanjani O., et al., 2020 [66]	26	Acholic stools or increase in serum bilirubin + fever or increase in inflammatory parameters	34.6%	-
Chen G., et al., 2021 [38]	180	1. Fever ≥ 38 °C or elevated CRP and 2. Recurrent acholic stool or jaundice with elevated bilirubin	66.1%	NLS, 84.4%
Chung P.H.Y., et al., 2021 [67]	231	Fever > 38.5 °C and bilirubin > 20 μmol/L on 2 consecutive blood samples; severe cholangitis if more than 2 weeks of antibiotics.	25.7%	NLS, 66.2%
Goh L., et al., 2021 [39]	54	1. Systemic inflammation: fever or elevated inflammatory markers CRP and WBC and 2 evidence of cholestasis or abnormal liver function tests—PA, GGT, AST, ALT > 1.5 normal ranges and/or elevation from baseline levels	72%	NLS, 79.4%

## References

[1] Nio M., Wada M., Sasaki H., Tanaka H., Okamura A. (2012). Risk factors affecting late-presenting liver failure in adult patients with biliary atresia. J. Pediatr. Surg..

[2] Koga H., Wada M., Nakamura H., Miyano G., Okawada M., Lane G.J., Okazaki T., Yamataka A. (2013). Factors influencing jaundice-free survival with the native liver in post-portoenterostomy biliary atresia patients: Results from a single institution. J. Pediatr. Surg..

[3] Chen S.Y., Lin C.C., Tsan Y.T., Chan W.C., Wang J.D., Chou Y.J., Lin C.H. (2018). Number of cholangitis episodes as a prognostic marker to predict timing of liver transplantation in biliary atresia patients after Kasai portoenterostomy. BMC Pediatr..

[4] Hung P.Y., Chen C.C., Chen W.J., Lai H.S., Hsu W.M., Lee P.H., Ho M.C., Chen T.H., Ni Y.H., Chen H.L. (2006). Long-term prognosis of patients with biliary atresia: A 25 year summary. J. Pediatr. Gastroenterol. Nutr..

[5] Li D., Chen X., Fu K., Yang J., Feng J. (2017). Preoperative nutritional status and its impact on cholangitis after Kasai portoenterostomy in biliary atresia patients. Pediatr. Surg. Int..

[6] Liu J., Dong R., Chen G., Dong K., Zheng S. (2019). Risk factors and prognostic effects of cholangitis after Kasai procedure in biliary atresia patients: A retrospective clinical study. J. Pediatr. Surg..

[7] Ryon E.L., Parreco J.P., Sussman M.S., Quiroz H.J., Willobee B.A., Perez E.A., Sola J.E., Thorson C.M. (2020). Drivers of Hospital Readmission and Early Liver Transplant after Kasai Portoenterostomy. J. Surg. Res..

[8] Decharun K., Leys C.M., West K.W., Finnell S.M. (2016). Prophylactic Antibiotics for Prevention of Cholangitis in Patients With Biliary Atresia Status Post-Kasai Portoenterostomy: A Systematic Review. Clin. Pediatr..

[9] Bu L.N., Chen H.L., Chang C.J., Ni Y.H., Hsu H.Y., Lai H.S., Hsu W.M., Chang M.H. (2003). Prophylactic oral antibiotics in prevention of recurrent cholangitis after the Kasai portoenterostomy. J. Pediatr. Surg..

[10] Wu E.T., Chen H.L., Ni Y.H., Lee P.I., Hsu H.Y., Lai H.S., Chang M.H. (2001). Bacterial cholangitis in patients with biliary atresia: Impact on short-term outcome. Pediatr. Surg. Int..

[11] Gunadi, Gunawan T.A., Widiyanto G., Yuanita A., Mulyani N.S., Makhmudi A. (2018). Liver transplant score for prediction of biliary atresia patients’ survival following Kasai procedure. BMC Res. Notes.

[12] Lee J.Y., Lim L.T., Quak S.H., Prabhakaran K., Aw M. (2014). Cholangitis in children with biliary atresia: Health-care resource utilisation. J. Paediatr. Child Health.

[13] Chuang J.H., Lee S.Y., Shieh C.S., Chen W.J., Chang N.K. (2000). Reappraisal of the role of the bilioenteric conduit in the pathogenesis of postoperative cholangitis. Pediatr. Surg. Int..

[14] Van Heurn L.W.E., Saing H., Tam P.K. (2003). Cholangitis after hepatic portoenterostomy for biliary atresia: A multivariate analysis of risk factors. J. Pediatr..

[15] Stagg H., Cameron B.H., Ahmed N., Butler A., Jimenez-Rivera C., Yanchar N.L., Martin S.R., Emil S., Anthopoulos G., Schreiber R.A. (2017). Variability of diagnostic approach, surgical technique, and medical management for children with biliary atresia in Canada—Is it time for standardization?. J. Pediatr. Surg..

[16] Wada K., Takada T., Kawarada Y., Nimura Y., Miura F., Yoshida M., Mayumi T., Strasberg S., Pitt H.A., Gadacz T.R. (2007). Diagnostic criteria and severity assessment of acute cholangitis: Tokyo Guidelines. J. Hepatobiliary Pancreat. Surg..

[17] Takada T., Strasberg S.M., Solomkin J.S., Pitt H.A., Gomi H., Yoshida M., Mayumi T., Miura F., Gouma D.J., Garden O.J. (2013). TG13: Updated Tokyo Guidelines for the management of acute cholangitis and cholecystitis. J. Hepatobiliary Pancreat. Sci..

[18] Lai H.S., Chen W.J., Chen C.C., Hung W.T., Chang M.H. (2006). Long-term prognosis and factors affecting biliary atresia from experience over a 25 year period. Chang Gung. Med. J..

[19] Pang W.B., Zhang T.C., Chen Y.J., Peng C.H., Wang Z.M., Wu D.Y., Wang K. (2019). Ten-Year Experience in the Prevention of Post-Kasai Cholangitis. Surg. Infect..

[20] Wong K.K., Fan A.H., Lan L.C., Lin S.C., Tam P.K. (2004). Effective antibiotic regime for postoperative acute cholangitis in biliary atresia—An evolving scene. J. Pediatr. Surg..

[21] Petersen C., Harder D., Melter M., Becker T., Wasielewski R.V., Leonhardt J., Ure B.M. (2008). Postoperative high-dose steroids do not improve mid-term survival with native liver in biliary atresia. Am. J. Gastroenterol..

[22] Graham B., Regehr G., Wright J.G. (2003). Delphi as a method to establish consensus for diagnostic criteria. J. Clin. Epidemiol..

[23] Dalkey N.C. (1963). An experimental application of the Delphi method to the use of experts. Manag. Sci..

[24] Burns K.E., Duffett M., Kho M.E., Meade M.O., Adhikari N.K., Sinuff T., Cook D.J., Group A. (2008). A guide for the design and conduct of self-administered surveys of clinicians. CMAJ.

[25] Lally K.P., Kanegaye J., Matsumura M., Rosenthal P., Sinatra F., Atkinson J.B. (1989). Perioperative factors affecting the outcome following repair of biliary atresia. Pediatrics.

[26] Meyers R.L., Book L.S., O’Gorman M.A., Jackson W.D., Black R.E., Johnson D.G., Matlak M.E. (2003). High-dose steroids, ursodeoxycholic acid, and chronic intravenous antibiotics improve bile flow after Kasai procedure in infants with biliary atresia. J. Pediatr. Surg..

[27] Kelly D.A., Davenport M. (2007). Current management of biliary atresia. Arch. Dis. Child..

[28] Stringer M.D., Davison S.M., Rajwal S.R., McClean P. (2007). Kasai portoenterostomy: 12-year experience with a novel adjuvant therapy regimen. J. Pediatr. Surg..

[29] Vejchapipat P., Passakonnirin R., Sookpotarom P., Chittmittrapap S., Poovorawan Y. (2007). High-dose steroids do not improve early outcome in biliary atresia. J. Pediatr. Surg..

[30] De Vries W., de Langen Z.J., Groen H., Scheenstra R., Peeters P.M., Hulscher J.B., Verkade H.J., Netherlands Study Group of Biliary Atresia and Registry (NeSBAR) (2012). Biliary atresia in the Netherlands: Outcome of patients diagnosed between 1987 and 2008. J. Pediatr..

[31] Wang B., Feng Q., Ye X., Zeng S. (2014). The experience and technique in laparoscopic portoenterostomy for biliary atresia. J. Laparoendosc. Adv. Surg. Tech. A.

[32] Tyraskis A., Davenport M. (2016). Steroids after the Kasai procedure for biliary atresia: The effect of age at Kasai portoenterostomy. Pediatr. Surg. Int..

[33] Webb N.L., Jiwane A., Ooi C.Y., Nightinghale S., Adams S.E., Krishnan U. (2017). Clinical significance of liver histology on outcomes in biliary atresia. J. Paediatr. Child Health.

[34] Lee W.S., Ong S.Y., Foo H.W., Wong S.Y., Kong C.X., Seah R.B., Ng R.T. (2017). Chronic liver disease is universal in children with biliary atresia living with native liver. World J. Gastroenterol..

[35] Parolini F., Boroni G., Milianti S., Tonegatti L., Armellini A., Garcia Magne M., Pedersini P., Torri F., Orizio P., Benvenuti S. (2019). Biliary atresia: 20-40-year follow-up with native liver in an Italian centre. J. Pediatr. Surg..

[36] Ramachandran P., Safwan M., Balaji M.S., Unny A.K., Akhtarkhavari A., Tamizhvanan V., Rela M. (2019). Early Cholangitis after Portoenterostomy in Children with Biliary Atresia. J. Indian Assoc. Pediatr. Surg..

[37] Baek S.H., Kang J.M., Ihn K., Han S.J., Koh H., Ahn J.G. (2020). The Epidemiology and Etiology of Cholangitis After Kasai Portoenterostomy in Patients With Biliary Atresia. J. Pediatr. Gastroenterol. Nutr..

[38] Chen G., Liu J., Huang Y., Wu Y., Lu X., Dong R., Shen Z., Sun S., Jiang J., Zheng S. (2021). Preventive effect of prophylactic intravenous antibiotics against cholangitis in biliary atresia: A randomized controlled trial. Pediatr. Surg. Int..

[39] Goh L., Phua K.B., Low Y., Chiang L.W., Yong C., Chiou F.K. (2021). Analysis of Cholangitis Rates with Extended Perioperative Antibiotics and Adjuvant Corticosteroids in Biliary Atresia. Pediatr. Gastroenterol. Hepatol. Nutr..

[40] Lien T.H., Bu L.N., Wu J.F., Chen H.L., Chen A.C., Lai M.W., Shih H.H., Lee I.H., Hsu H.Y., Ni Y.H. (2015). Use of Lactobacillus casei rhamnosus to Prevent Cholangitis in Biliary Atresia After Kasai Operation. J. Pediatr. Gastroenterol. Nutr..

[41] Chiang L.W., Lee C.Y., Krishnaswamy G., Nah S.A., Kader A., Ong C., Low Y., Phua K.B. (2017). Seventeen years of Kasai portoenterostomy for biliary atresia in a single Southeast Asian paediatric centre. J. Paediatr. Child Health.

[42] Li Z., Ye Y., Wu Z., Wang B. (2017). Learning Curve Analysis of Laparoscopic Kasai Portoenterostomy. J. Laparoendosc. Adv. Surg. Tech. A.

[43] Calinescu A.M., Wilde J.C.H., Korff S., McLin V.A., Wildhaber B.E. (2020). Perioperative Complications after Kasai Hepatoportoenterostomy: Data from the Swiss National Biliary Atresia Registry. Eur. J. Pediatr. Surg..

[44] Chung P.H.Y., Chok K.S.H., Wong K.K.Y., Tam P.K.H., Lo C.M. (2020). Determining the optimal timing of liver transplant for pediatric patients after Kasai portoenterostomy based on disease severity scores. J. Pediatr. Surg..

[45] Sifri C.D., Madoff L.C. (2015). Infections of the Liver and Biliary system (Liver abscess, cholangitis, cholecystitis). Mandell, Douglas and Bennett’s Priciples and Practice of Infectious Diseases.

[46] Kumagi T., Drenth J.P., Guttman O., Ng V., Lilly L., Therapondos G., Hiasa Y., Michitaka K., Onji M., Watanabe Y. (2012). Biliary atresia and survival into adulthood without transplantation: A collaborative multicentre clinic review. Liver Int..

[47] Kiriyama S., Takada T., Hwang T.L., Akazawa K., Miura F., Gomi H., Mori R., Endo I., Itoi T., Yokoe M. (2017). Clinical application and verification of the TG13 diagnostic and severity grading criteria for acute cholangitis: An international multicenter observational study. J. Hepatobiliary Pancreat. Sci..

[48] Chung P.H.Y., Tam P.K.H., Wong K.K.Y. (2018). Does the identity of the bacteria matter in post-Kasai cholangitis? A comparison between simple and intractable cholangitis. J. Pediatr. Surg..

[49] Sandy N.S., Hessel G., Bellomo-Brandao M.A. (2020). Major Complications of Pediatric Percutaneous Liver Biopsy Do Not Differ Among Physicians with Different Degrees of Training. Am. J. Gastroenterol..

[50] Luo Y., Zheng S. (2008). Current concept about postoperative cholangitis in biliary atresia. World J. Pediatr..

[51] Pakarinen M.P., Rintala R.J. (2011). Surgery of biliary atresia. Scand. J. Surg..

[52] Selvalingam S., Mahmud M.N., Thambidorai C.R., Zakaria Z., Mohan N., Isa, Sheila M. (2002). Jaundice clearance and cholangitis in the first year following portoenterostomy for biliary atresia. Med. J. Malays..

[53] Ogasawara Y., Yamataka A., Tsukamoto K., Okada Y., Lane G.J., Kobayashi H., Miyano T. (2003). The intussusception antireflux valve is ineffective for preventing cholangitis in biliary atresia: A prospective study. J. Pediatr. Surg..

[54] Kobayashi H., Yamataka A., Koga H., Okazaki T., Tamura T., Urao M., Yanai T., Lane G.J., Miyano T. (2005). Optimum prednisolone usage in patients with biliary atresia postportoenterostomy. J. Pediatr. Surg..

[55] Shinohara T., Muraji T., Tsugawa C., Nishijima E., Satoh S., Takamizawa S. (2005). Efficacy of urinary sulfated bile acids for diagnosis of bacterial cholangitis in biliary atresia. Pediatr. Surg. Int..

[56] Sanghai S.R., Shah I., Bhatnagar S., Murthy A. (2009). Incidence and prognostic factors associated with biliary atresia in western India. Ann. Hepatol..

[57] Suzuki T., Hashimoto T., Kondo S., Sato Y., Hussein M.H. (2010). Evaluating patients’ outcome post-Kasai operation: A 19-year experience with modification of the hepatic portoenterostomy and applying a novel steroid therapy regimen. Pediatr. Surg. Int..

[58] Ng V.L., Haber B.H., Magee J.C., Miethke A., Murray K.F., Michail S., Karpen S.J., Kerkar N., Molleston J.P., Romero R. (2014). Medical status of 219 children with biliary atresia surviving long-term with their native livers: Results from a North American multicenter consortium. J. Pediatr..

[59] Wada M., Nakamura H., Koga H., Miyano G., Lane G.J., Okazaki T., Urao M., Murakami H., Kasahara M., Sakamoto S. (2014). Experience of treating biliary atresia with three types of portoenterostomy at a single institution: Extended, modified Kasai, and laparoscopic modified Kasai. Pediatr. Surg. Int..

[60] Qiao G., Li L., Cheng W., Zhang Z., Ge J., Wang C. (2015). Conditional probability of survival in patients with biliary atresia after Kasai portoenterostomy: A Chinese population-based study. J. Pediatr. Surg..

[61] Kelay A., Davenport M. (2017). Long-term outlook in biliary atresia. Semin. Pediatr. Surg..

[62] Jiang H., Gao P., Chen H., Zhong Z., Shu M., Zhang Z., She J., Liu J. (2018). The Prognostic Value of CD8(+) and CD45RO(+) T Cells Infiltration and Beclin1 Expression Levels for Early Postoperative Cholangitis of Biliary Atresia Patients after Kasai Operation. J. Korean Med. Sci..

[63] Nakajima H., Koga H., Okawada M., Nakamura H., Lane G.J., Yamataka A. (2018). Does time taken to achieve jaundice-clearance influence survival of the native liver in post-Kasai biliary atresia?. World J. Pediatr..

[64] Xiao H., Huang R., Chen L., Diao M., Li L. (2018). The Application of a Shorter Loop in Kasai Portoenterostomy Reconstruction for Ohi Type III Biliary Atresia: A Prospective Randomized Controlled Trial. J. Surg. Res..

[65] Ginstrom D.A., Hukkinen M., Kivisaari R., Pakarinen M.P. (2019). Biliary Atresia-associated Cholangitis: The Central Role and Effective Management of Bile Lakes. J. Pediatr. Gastroenterol. Nutr..

[66] Madadi-Sanjani O., Schukfeh N., Uecker M., Eckmann S., Dingemann J., Ure B.M., Petersen C., Kuebler J.F. (2021). The Intestinal Flora at Kasai Procedure in Children with Biliary Atresia Appears Not to Affect Postoperative Cholangitis. Eur. J. Pediatr. Surg..

[67] Chung P.H.Y., Chan E.K.W., Yeung F., Chan A.C.Y., Mou J.W.C., Lee K.H., Hung J.W.S., Leung M.W.Y., Tam P.K.H., Wong K.K.Y. (2021). Life long follow up and management strategies of patients living with native livers after Kasai portoenterostomy. Sci. Rep..

