# Supplementary material for: Cholangitis Definition and Treatment after Kasai Hepatoportoenterostomy for Biliary Atresia: A Delphi Process and International Expert Panel
Source: J Clin Med. 2022 Jan 19;11(3):494. doi: 10.3390/jcm11030494 (PMC8836553; doi:10.3390/jcm11030494)
Supplement: Supplementary file 1 [file jcm-11-00494-s001.zip › Supplementary Document S1.pdf]

Cholangitis definition and management after Kasai hepatoportoenterostomy for biliary atresia

INTRODUCTION

Acute cholangitis is the most feared complication after Kasai hepatoportoenterostomy for biliary atresia due to its negative impact on prognosis, repeated bouts of cholangitis decreasing patient survival. In the absence of standard criteria for diagnosis of acute cholangitis after Kasai hepatoportoenterostomy, practical management guidelines have never been established. We believe that this study will allow for a more detailed insight in current practices and will help to define cholangitis and antibiotic prophylaxis/treatment policies after Kasai hepatoportoenterostomy. Your confidential responses to this questionnaire are very important to us.

Please note that you are eligible for this survey if you manage biliary atresia patients after Kasai hepatoportoenterostomy.

This questionnaire will take you about 10 minutes to complete.

If you have any questions, please don't hesitate to contact

Dr Ana Calinescu
ana-maria.calinescu@hcuge.ch

or

Dr Madadi-Sanjani
Madadi-Sanjani.Omid@mh-hannover.de

We thank you in advance for your much-appreciated collaboration.

CURRENT PRACTICE

Cholangitis definition

Q1 Which clinical signs following Kasai hepatoportoenterostomy for biliary atresia do you use for cholangitis diagnosis?

- Fevers / Shivering
- Stool colour change
- New or increasing jaundice
- Abdominal distension
- Other

Q2 **Which clinical symptoms following Kasai hepatoportoenterostomy for biliary atresia do you use for cholangitis diagnosis?**

- Abdominal pain
- Other

Q2 Which laboratory tests do you include in your cholangitis diagnosis?

- Elevated white blood cell counts
- Elevated CRP
- Elevated Interleukin-6
- Elevated Procalcitonin
- Positive blood cultures
- Elevated / increasing bilirubin
- Elevated/ increasing GGT
- Elevated/ increasing transaminases
- Positive cultures of jejunal limb
- Other

Q3 Do you use imaging techniques for diagnosis of cholangitis?

- Yes
- No

Q4 If yes, please specify:

- Ultrasound
- CT
- MRI/ MRCP
- HBSS
- Other

Q5 Which findings during imaging do you consider compatible with cholangitis?

- o Biliary dilatation
- o Other

Q6 Do you use liver biopsy for cholangitis diagnosis?

- o Yes
- o No

Q7 The combination of which items have to apply, for cholangitis diagnosis?

- o Fevers / Shivering
- o New or increasing jaundice
- o Stool colour change
- o Abdominal distension
- o Abdominal pain
- o Elevated white blood counts
- o Elevated CRP
- o Elevated Interleukin-6
- o Elevated Procalcitonin
- o Positive blood cultures
- o Positive cultures of the jejunal limb
- o Elevated / increasing bilirubin
- o Elevated/ increasing GGT
- o Elevated/ increasing transaminases
- o Biliary dilatation
- o Positive liver pathology
- o Other

Steroids

Q1 Do you use steroids in the postoperative period after Kasai hepatoportoenterostomy for biliary atresia?

- o Yes
- o No

Q2 If yes, please specify:

- o Type of steroid given: …
- o What is the precise dosage: …
- o Duration of administration: …
- o Starting from postoperative day: …
- o Type of administration: …

Antibiotic prophylaxis

Q1 Do you use antibiotic prophylaxis for cholangitis after Kasai hepatoportoenterostomy for biliary atresia?

- Yes
- No

Q2 The antibiotic(s) used for cholangitis prophylaxis after Kasai hepatoportoenterostomy for biliary atresia is (are) given

- To all patients
- Only if partial bile flow (please give your definition of partial bile flow …)
- Only if no bile flow (please give your definition of no bile flow …)
- Only if intrahepatic bile duct cystic dilatations
- Only if other: ... (please specify)

Q3 Which antibiotic(s) do you use for cholangitis prophylaxis after Kasai hepatoportoenterostomy for biliary atresia?

- Ceftriaxone
- Cefixime
- Other cephalosporin: ... (please specify)
- Gentamicin
- Neomycin
- Other aminoglycoside: ... (please specify)
- Trimethoprim and Sulfamethoxazole
- Amoxicillin and clavulanic acid
- Metampicillin
- Minocycline
- Ciprofloxacin
- Ofloxacin
- Imipenem
- Other antibiotic: ... (please specify)

Q4 When do you start using it?

- Intraoperative
- Postoperative Day 1
- Other: ... (please specify)

Q5 For how long do you use it?

- Single dose
- 24h
- 48h
- 5 days
- 7 days
- 10 days

- 14 days
- 21 days
- Other: ... (please specify)

Q6 What is the dose that you use?

- Please specify: …

Q7 How do you administrate it?

- Intravenous injection
- Intramuscular injection
- Oral administration

Q8 When you stop it, do you switch to another antibiotic?

- Yes
- No

Q9 If yes, to what antibiotic(s)?

- Please specify: …

Q10 If yes, for how long?

- Please specify: …

Q11 If yes, how do you administrate the new antibiotic?

- Intravenous injection
- Intramuscular injection
- Oral administration

Q12 What makes you change it?

- In all patients, according to local protocole
- Depending on the results from fluid cultures from the tip of the jejunal limb at bile duct reconstruction
- After the 1st episode of cholangitis
- Recurrent cholangitis (please specify recurrent …)
- Depending on the results from blood cultures, if drawn
- Other: ... (please specify)

Cholangitis treatment

Q1 The antibiotic(s) used for cholangitis treatment after Kasai hepatoportoenterostomy for biliary atresia is (are) given generally:

- 1 week

- 10 days
- 2 weeks
- 3 weeks
- Other

Q2 Which antibiotic(s) do you use for cholangitis treatment after Kasai hepatoportoenterostomy for biliary atresia?

- Ceftriaxone
- Cefixime
- Other cephalosporin: ... (please specify)
- Gentamicin
- Neomycin
- Other aminoglycoside: ... (please specify)
- Trimethoprim and Sulfamethoxazole
- Amoxicillin and clavulanic acid
- Metampicillin
- Minocycline
- Ciprofloxacin
- Ofloxacin
- Imipenem
- Other antibiotic: ... (please specify)

Q3 How do you administrate it?

- Intravenous injection
- Intramuscular injection
- Oral administration

Q4 When you stop it, do you switch to another antibiotic?

- Yes
- No

Q5 If yes, to what antibiotic(s)?

- Please specify: …

Q6 If yes, for how long?

- Please specify: …

Q7 If yes, how do you administrate it?

- Intravenous injection
- Intramuscular injection
- Oral administration

Q8 What makes you change it?

- In all patients, according to local protocole

- Depending on the results from fluid cultures from the tip of the jejunal limb at bile duct reconstruction
- After the 1st episode of cholangitis
- Recurrent cholangitis (please specify recurrent …)
- Bile lakes
- Hepatholithiasis
- Depending on the results from blood cultures, if drawn
- Other: ... (please specify)

DEMOGRAPHIC DATA

Q1 What is your gender?

- Female
- Male

Q2 What is your age?

- 25-29 years
- 30-34 years
- 35-39 years
- 40-44 years
- 45-49 years
- 50-54 years
- 55-59 years
- 60-64 years
- 65 years and more

Q3 What is your primary language?

- Bulgarian
- Czech
- Chinese
- Danish
- Dutch
- English
- Finnish
- French
- German
- Greek
- Italian
- Japanese
- Polish
- Portuguese
- Slovak
- Spanish
- Swedish
- Other (please specify): …

Q4 In what country do you practice Kasai hepatoportoenterostomy?

- Australia
- Austria
- Belgium
- Bulgaria
- Canada
- China
- Czech republic
- Denmark
- Finland
- France
- Germany
- Greece
- India
- Israel
- Italy
- Japan
- Netherlands
- New Zeeland
- Poland
- Portugal
- Slovakia
- Spain
- Sweden
- Switzerland
- United Kingdom
- United States of America
- Other (please specify): …

Q5 Is your center specialized in caring for the biliary atresia patients?

- Yes
- No

Q6 What is your academic rank?

- Full professor
- Associate professor
- Assistant professor
- Lecturer
- Fellow in transplant surgery
- Resident
- No academic affiliation
- Other (please specify): …

Q7 Year of graduation when you obtained your MD degree?

- Prior to 1970
- 1970-1979

- 1980-1989
- 1990-1999
- 2000-2009

Q8 Number of years since you perform the Kasai hepatoportoenterostomy?

- 0-4 years
- 5-9 years
- 10-14 years
- 15 years or more

Q9 How many Kasai hepatoportoenterostomies have you done in the last 12 months?

- less than 10
- 10 – 20
- 20 – 30
- 30 – 40
- more than 40

Q10 Please indicate the average number of Kasai hepatoportoenterostomies performed per year in your center:

- less than 10
- 10 – 20
- 20 – 30
- 30 – 40
- more than 40

Q11 What is the percentage of cholangitis after Kasai hepatoportoenterostomy, since you have introduced the actual antibiotic prophylaxis after Kasai hepatoportoenterostomy?

- 1-10%
- 10-20%
- 20-30%
- 30-40%
- 40-50%
- 50-60%
- 60-70%
- 70-80%
- 80-90%
- More than 90%
- If known, exact number: …

If you wish to be informed about the results of this survey please enter your e-mail address.
