# Supplementary material for: Cholangitis Definition and Treatment after Kasai Hepatoportoenterostomy for Biliary Atresia: A Delphi Process and International Expert Panel
Source: J Clin Med. 2022 Jan 19;11(3):494. doi: 10.3390/jcm11030494 (PMC8836553; doi:10.3390/jcm11030494)
Supplement: Supplementary file 1 [file jcm-11-00494-s001.zip › Supplementary Document S2.pdf]

Definition of cholangitis and management after Kasai hepatoportoenterostomy for biliary atresia – phase II

INTRODUCTION

Acute cholangitis after Kasai hepatoportoenterostomy is frequent. Yet, there exists no standardized definition nor management guidelines. After a first round of definition and management questions we would be very grateful if you could fill in this 2nd questionnaire to help us define cholangitis and prophylaxis/treatment policies after Kasai hepatoportoenterostomy for babies with biliary atresia.

Please note that you are eligible for this survey if you manage biliary atresia patients after Kasai hepatoportoenterostomy. This questionnaire will take you about 5 minutes to complete. If you have any questions, please don't hesitate to contact

- Dr Ana Calinescu, Ana-maria.Calinescu@hcuge.ch

or

- Dr Madadi-Sanjani, Madadi-Sanjani.Omid@mh-hannover.de

Your confidential responses to this questionnaire are very important to us. The current literature as well as your precious input will be discussed at BARD in Bruge in July 2020, with the aim to come up with a Consensus.

We thank you in advance for your much-appreciated collaboration.

Ana Calinescu
Omid Madadi-Sanjani
Claus Peterson
Barbara Wildhaber

<u>Cholangitis definition</u>

This part of the questionnaire treats the definition of cholangitis after Kasai hepatoportoenterostomy for biliary atresia.

Q1 Rate the following elements according to their importance for your diagnosis of cholangitis after a Kasai:

Please rate it from 1 (completely unimportant) to 10 (extremely important)

- **fever and/or shivering**
- **stool colour change (discoloration)**
- **new or increasing jaundice**
- **abdominal discomfort**
- **inflammatory response (white blood cell count and/or CRP and/or procalcitonin)**
- **bile lakes diagnosed on ultrasound**
- **increased/increasing transaminases**
- **increased/increasing GGT and/or bilirubin**
- **positive blood cultures**

Q2 For you to <u>suspect</u> a cholangitis after a Kasai, the mandatory presence of following elements might be:

A. **Clinical elements**
    a. **Fever and or shivering**
    b. **Stool colour change**
    c. **New or increasing jaundice**
    d. **Abdominal discomfort**
B. **Laboratory and imagining elements**
    a. **Inflammatory response (WBC and/or CRP and/or procalcitonin)**
    b. **Increased/increasing transaminases**
    c. **Increased/increasing GGT and/or bilirubin**
    d. **Bile lakes**

Please choose the combination of elements to define a <u>suspected</u> cholangitis:

- o 1 or more elements from A
- o 2 or more elements from A
- o 3 or more elements from A
- o All 4 elements from A
- o 1 of the 4 elements from A and 1 of the 4 elements from B
- o 1 of the 4 elements from A and 2 of the 4 elements from B
- o 1 of the 4 elements from A and 3 of the 4 elements from B
- o 1 of the 4 elements from A and all 4 elements from B
- o 2 of the 4 elements from A and 1 of the 4 elements from B
- o 2 of the 4 elements from A and 2 of the 4 elements from B
- o 2 of the 4 elements from A and 3 of the 4 elements from B
- o 2 of the 4 elements from A and all 4 elements from B
- o 3 of the 4 elements from A and 1 of the 4 elements from B
- o 3 of the 4 elements from A and 2 of the 4 elements from B

- 3 of the 4 elements from A and 3 of the 4 elements from B
- 3 of the 4 elements from A and all 4 elements from B
- All 4 elements from A and 1 of the 4 element from B
- All 4 elements from A and 2 of the 4 elements from B
- All 4 elements from A and 3 the 4 elements from B
- All 4 elements from A and all 4 elements from B

Q11 For you to define a <u>confirmed</u> cholangitis after a Kasai, the mandatory presence of following elements might be:

A. clinical elements
  a. Fever and or shivering
  b. Stool colour change
  c. New or increasing jaundice
  d. Abdominal discomfort
B. laboratory and imagining elements
  a. Inflammatory response (WBC and/or CRP and/or procalcitonin)
  b. Increased/increasing transaminases
  c. Increased/increasing GGT and/or bilirubin
  d. Bile lakes

Please choose the combination of elements to define a <u>confirmed</u> cholangitis:

- 1 or more elements from A
- 2 or more elements from A
- 3 or more elements from A
- All 4 elements from A
- 1 of the 4 elements from A and 1 of the 4 elements from B
- 1 of the 4 elements from A and 2 of the 4 elements from B
- 1 of the 4 elements from A and 3 of the 4 elements from B
- 1 of the 4 elements from A and all 4 elements from B
- 2 of the 4 elements from A and 1 of the 4 elements from B
- 2 of the 4 elements from A and 2 of the 4 elements from B
- 2 of the 4 elements from A and 3 of the 4 elements from B
- 2 of the 4 elements from A and all 4 elements from B
- 3 of the 4 elements from A and 1 of the 4 elements from B
- 3 of the 4 elements from A and 2 of the 4 elements from B
- 3 of the 4 elements from A and 3 of the 4 elements from B
- 3 of the 4 elements from A and all 4 elements from B
- All 4 elements from A and 1 of the 4 elements from B
- All 4 elements from A and 2 of the 4 elements from B
- All 4 elements from A and 3 the 4 elements from B
- All 4 elements from A and all 4 elements from B

<u>Antibiotic prophylaxis</u>

This part of the questionnaire treats the use of antibiotics in a normal, standard postoperative setting after a Kasai hepatoportoenterostomy.

Q1 For how long do you keep <u>intravenous</u> antibiotic prophylaxis following a Kasai?

- None
- 1 week or less
- 10 days
- 2 weeks
- 3 weeks
- 1 month
- 3 months
- 6 months
- Other: please specify

Q1 For how long do you keep <u>peroral</u> antibiotic prophylaxis following a Kasai?

- None
- 1 week or less
- 10 days
- 2 weeks
- 3 weeks
- 1 month
- 3 months
- 6 months
- 1 year
- Other: please specify

Cholangitis treatment

This part of the questionnaire treats the use of antibiotics in case of a suspected (Q1) or confirmed (Q2) cholangitis at *any* time after the Kasai hepatoportoenterostomy.

Q1 For how long do you give intravenous antibiotic treatment for a <u>suspected</u> cholangitis after a Kasai?

- 3 days
- 1 week
- 2 weeks
- 3 weeks
- 1 month

Q2 For how long would you give intravenous antibiotic treatment for a <u>confirmed</u> cholangitis following Kasai hepatoportoenterostomy for biliary atresia?

- 1 week
- 10 days
- 3 weeks
- 1 month
- 3 months
- 6 months
- 1 year

Q3 After how many cholangitis episodes do you give long term cholangitis prophylaxis?

- 1

- 2
- 3
- 4
- 5
